# sRAGE alleviates neutrophilic asthma by blocking HMGB1/RAGE signalling in airway dendritic cells

**DOI:** 10.1038/s41598-017-14667-4

**Published:** 2017-10-27

**Authors:** Fang Zhang, Xin Su, Gang Huang, Xiao-Feng Xin, E-Hong Cao, Yi Shi, Yong Song

**Affiliations:** 1 Department of Pulmonary Medicine, Jinling Hospital, Nanjing University School of Medicine, Nanjing, China; 2 Department of Medical Genetics, The Third Military Medical University, Chongqing, China

## Abstract

Receptor for advanced glycation end products (RAGE) plays a role in inflammatory reactions. The soluble form of RAGE (sRAGE) acts as a decoy to inhibit interactions of RAGE with advanced glycation end products such as High mobility group box 1 (HMGB1). We have demonstrated that HMGB1 directs Th17 skewing by regulating dendritic cell (DC) functions in a previous study. However, the protective effects of HMGB1 blockade with sRAGE in the development of neutrophilic asthma remain unclear. Here, we showed that allergen challenge decreased expression of sRAGE in a murine model of neutrophilic asthma, correlating well with neutrophil counts and interleukin (IL)-17 production. When HMGB1 signalling was blocked by intratracheal administration of sRAGE before sensitisation, HMGB1 expression, neutrophilic inflammation, and Th17-type responses were reduced significantly. Anti-asthma effects of sRAGE were achieved by inhibition of RAGE and IL-23 expression in airway CD11c^+^ antigen-presenting cells. Finally, we showed that sRAGE inhibited Th17 polarisation induced by recombinant HMGB1 (rHMGB1)-activated dendritic cells (DCs) *in vitro*. Adoptive transfer of rHMGB1-activated DCs was sufficient to restore airway inflammation, whereas transfer of rHMGB1 plus sRAGE-activated DCs significantly reduced neutrophilic inflammation. Thus, sRAGE prevents Th17-mediated airway inflammation in neutrophilic asthma at least partly by blocking HMGB1/RAGE signalling in DCs.

## Introduction

Allergic asthma is one of the most common airway diseases worldwide^1^. Asthma is generally considered as an airway inflammatory disease characterised by obstruction due to the stimulation of environmental antigens. Despite the progress in therapies and determining the mechanism of pathogenesis in recent years, clinical control of asthma remains poor, which is mainly attributed to the high incidence of recurrence, exacerbation, and therapy resistance^2^. Increasing evidence suggests that asthma is a heterogeneous disorder regulated by distinct molecular mechanisms. T helper type (Th) 2 cells have been described in the pathogenesis of eosinophilic asthma^3^, whereas Th17 cells are mainly involved in severe asthma in which neutrophils contribute more than eosinophils to the inflammation. Although Th17 cells are involved in the development of steroid-refractory and neutrophilic asthma^4^, the underlying molecular mechanism responsible for the dysregulated immune responses mediated by Th17 cells still remains unclear. Therefore, it is of great importance to perform further studies regarding the molecular mechanism of Th17 polarisation in neutrophilic asthma.

Dendritic cells (DCs) are considered as the most important antigen-presenting cells (APCs) that are responsible for cytokine polarisation and expansion of Th2/Th17 subsets^5^. Many lines of evidence shows that cytokines interleukin (IL)-23 and IL-6 are required for DC-mediated Th17 cell differentiation and maintain cytokine expression in effector Th17 cells^6–8^. High mobility group box 1 (HMGB1) is a chromatin-associated protein. The function of HMGB1 is complicated and related to its cellular localisation^9^. In the nucleus, HMGB1 binds to DNA and aids transcription and DNA repair by directly binding to DNA and altering its conformation^10^. Nevertheless, HMGB1 can be released into the extracellular environment in response to certain stimulations, such as endotoxin^11^, and plays a critical role in the development of various diseases such as asthma, diabetes, neurodegeneration, and cancer^12–15^. Some studies postulate that increased HMGB1 enhances Th17 cell polarisation in chronic asthma, acute allograft rejection, and chronic infectious diseases^16–18^. Our previous study has demonstrated that HMGB1 directs Th17 skewing by regulating DC functions via IL-23 secretion, and blocking HMGB1 by an antibody may benefit attenuation of neutrophilic airway inflammation in asthma^19^.

Receptor for advanced glycation end products (RAGE), which is an important endogenous pattern recognition receptor, is a central cell surface receptor for HMGB1. RAGE has also been implicated in mediating the cytokine activity of HMGB1, which is a late inflammatory mediator of sepsis, and is involved in the host response to injury, infection, and inflammation^20^. RAGE exists as both membrane-bound and soluble receptors. Soluble RAGE (sRAGE) is generated through proteolytic cleavage of the extracellular domain of the cell surface receptor or through alternative RNA splicing^21,22^. Classically, sRAGE functions as a natural antagonist of RAGE signalling, because it sequesters RAGE ligands and inhibits RAGE-dependent cellular responses^23,24^. Previous studies have found that systemic sRAGE decreases significantly in subjects with neutrophilic asthma^25^, and correlates with the clinical severity of bronchial asthma and functionally in asthmatic children^26^. However, the molecular mechanisms of sRAGE deficiency in neutrophilic asthma are not yet fully understood. In this study, we evaluated whether sRAGE blocks HMBG1 signalling in DCs and subsequently contributes to alleviating Th17 polarisation in neutrophilic asthma, indicating that the HMBG1-RAGE axis might play a significant role in the development of neutrophilic asthma.

## Results

### Levels of sRAGE decrease significantly in bronchoalveolar lavage fluid (BALF) of asthmatic mice

**Figure 1:**
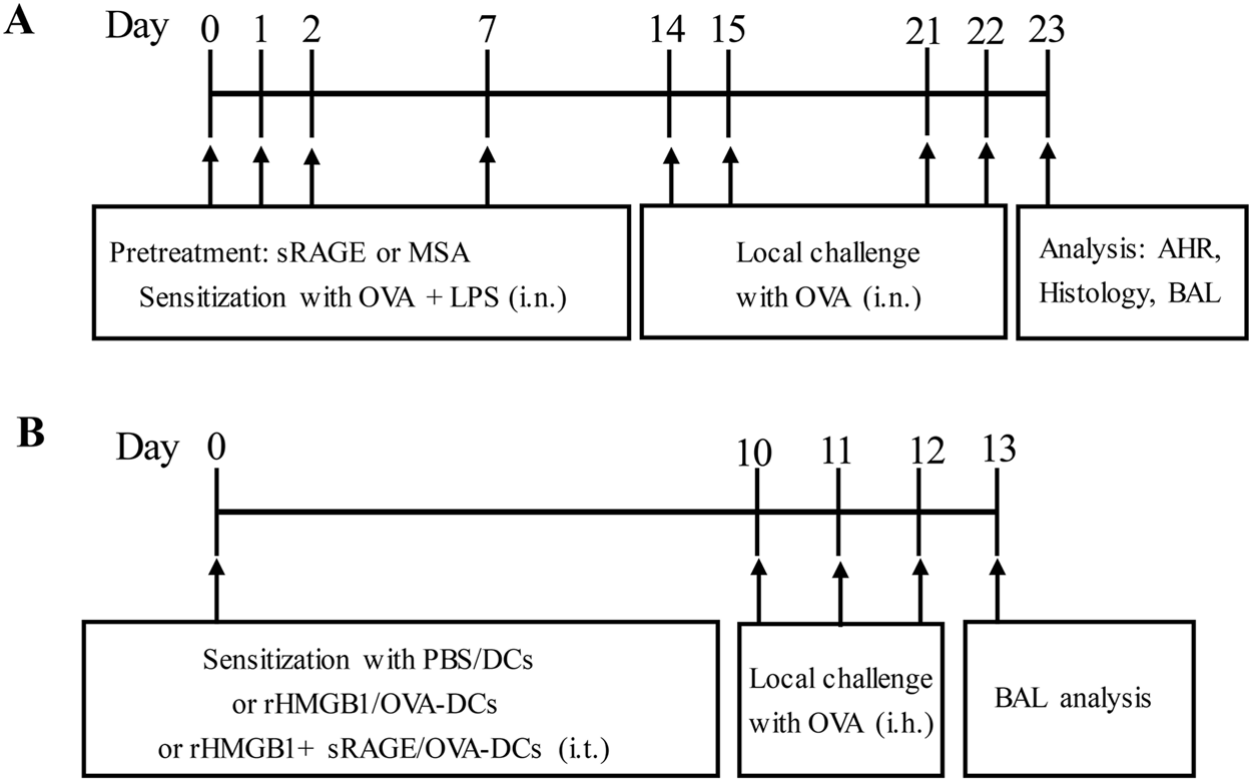
(**A**) Protocols for the OVA and LPS-induced murine model of neutrophilic asthma and experimental intervention. Mice were intranasally sensitised with OVA plus LPS on days 0, 1, 2, and 7 and then challenged with OVA on days 14, 15, 21, and 22. sRAGE or MSA was intranasally administered at 30 min before each OVA challenge. One day after the final challenge, the mice were sacrificed for further analyses. (**B**) Protocols for establishment of the murine model of asthma by the transfer of DCs and experimental intervention. Mice were intratracheally sensitised with PBS/DCs, HMGB1/OVA-DCs, or HMGB1 + sRAGE/OVA-DCs on day 0 and then challenged with OVA on days 10, 11, and 12. One day after the final challenge, the mice were sacrificed for further analyses.

**Figure 2:**
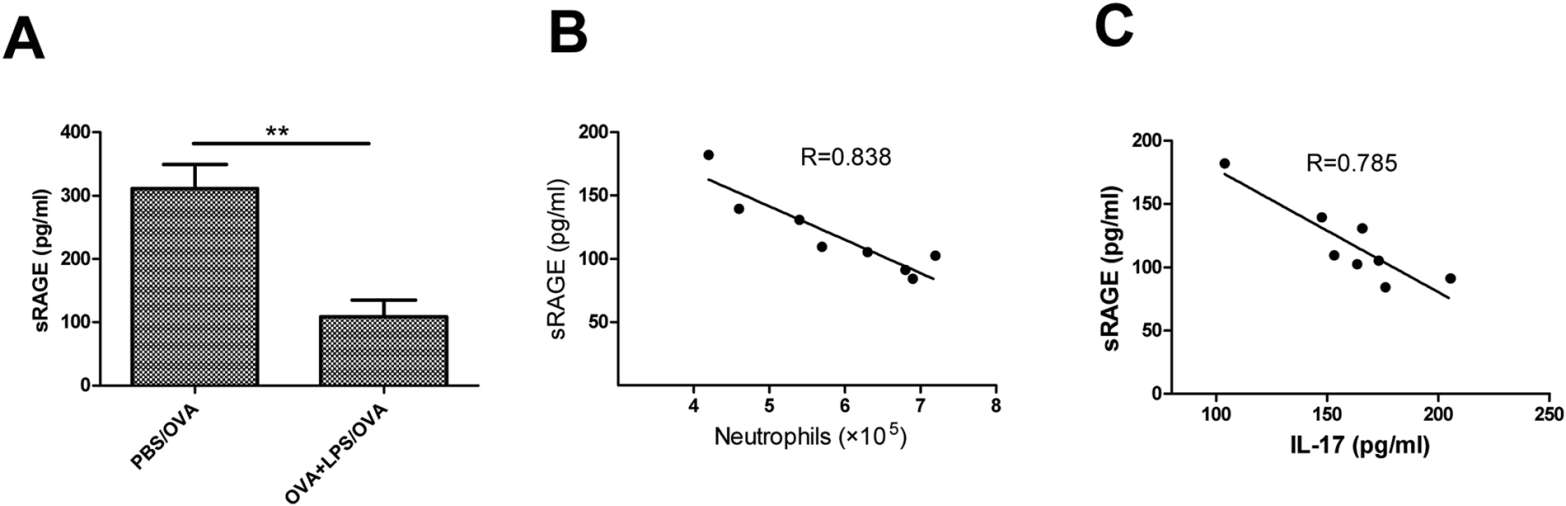
Levels of sRAGE decrease during asthmatic airway inflammation in the mouse model of neutrophilic asthma. (**A**) sRAGE levels analysed by ELISA in BALF of OVA plus LPS- or PBS-sensitised mice. (**B**) Negative correlation between the levels of sRAGE and neutrophils (r = 0.838), and (**C**) relatively lower correlation between sRAGE and IL-17 levels (r = 0.785) in BALF from OVA plus LPS-sensitised mice. Data are the mean ± SEM; n = 8 mice in each group.

Using a mouse model of neutrophilic asthma induced by ovalbumin (OVA) plus lipopolysaccharide (LPS) sensitisation followed by three sequential daily OVA challenges (Fig. 1A), we investigated the functional relevance of sRAGE levels and allergic Th17 responses in the disease. As shown in Fig. 2A, there was a significant decrease of sRAGE levels in BALF from mice sensitised with OVA plus LPS compared with phosphate-buffered saline (PBS)-sensitised controls. Moreover, a decrease of sRAGE in OVA plus LPS-sensitised mice but not in PBS-sensitised mice, correlated well with a rise in BALF neutrophils and IL-17 levels (Fig. 2B,C), suggesting a strong association of sRAGE with the pathogenesis of experimental neutrophilic asthma.

### sRAGE inhibits HMGB1 expression in lung tissue

**Figure 3:**
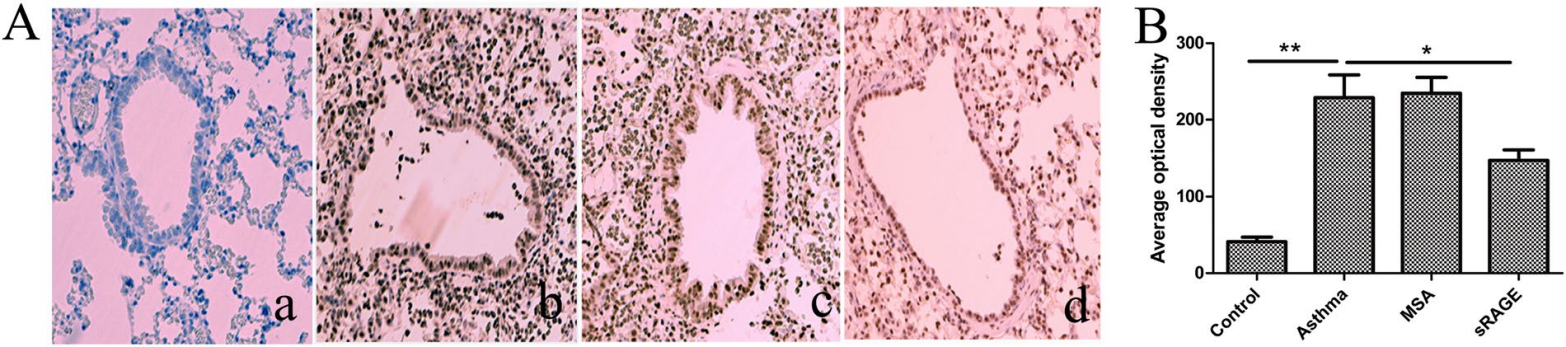
Local administration of sRAGE down-regulates HMGB1 expression in the lung. (**A**) Representative images of immunohistochemistry for HMGB1 in lung tissue from mice in Control (a), Asthma (b), MSA-treated (c), and sRAGE-treated groups (d). (**B**) The average optical density in the histogram represents HMGB1 expression. Values represent the means ± SEM (n = 5–6). *P < 0.05 and **P < 0.01 compared with the Asthma group.

Because HMGB1 has been identified as a crucial advanced glycation end product (AGE) that is involved in many inflammatory diseases, we considered that sRAGE may ameliorate airway inflammation via blockade of HMGB1 signalling. Using an HMGB1 immunostaining technique, we found that HMGB1 expression in lung tissue was elevated significantly in OVA and LPS-sensitised mice. However, this effect was inhibited by administration of sRAGE before sensitisation (Fig. 3A), suggesting that sRAGE has an inhibitory effect on HMGB1 expression. HMGB1 protein and mRNA levels in lung tissue were also measured by western blotting and quantitative PCR, respectively. The results showed that HMGB1 mRNA and protein levels were significantly decreased in sRAGE-treated mice compared with mouse serum albumin (MSA)-treated mice (Supplementary Fig. S1).

To eliminate the possibility of sRAGE masking the HMGB1 antibody epitope, two doses of sRAGE (200 or 400 ng/ml) were directly added to the lung homogenates of asthmatic mice, and then HMGB1 expression was evaluated using the HMGB1 antibody for western blotting. The results showed that the addition of sRAGE to lung homogenates had no effect on HMGB1 expression (Supplementary Fig. S1B), suggesting that sRAGE does not mask the HMGB1 antibody epitope.

### Local treatment with sRAGE reduces neutrophilic airway inflammation

**Figure 4:**
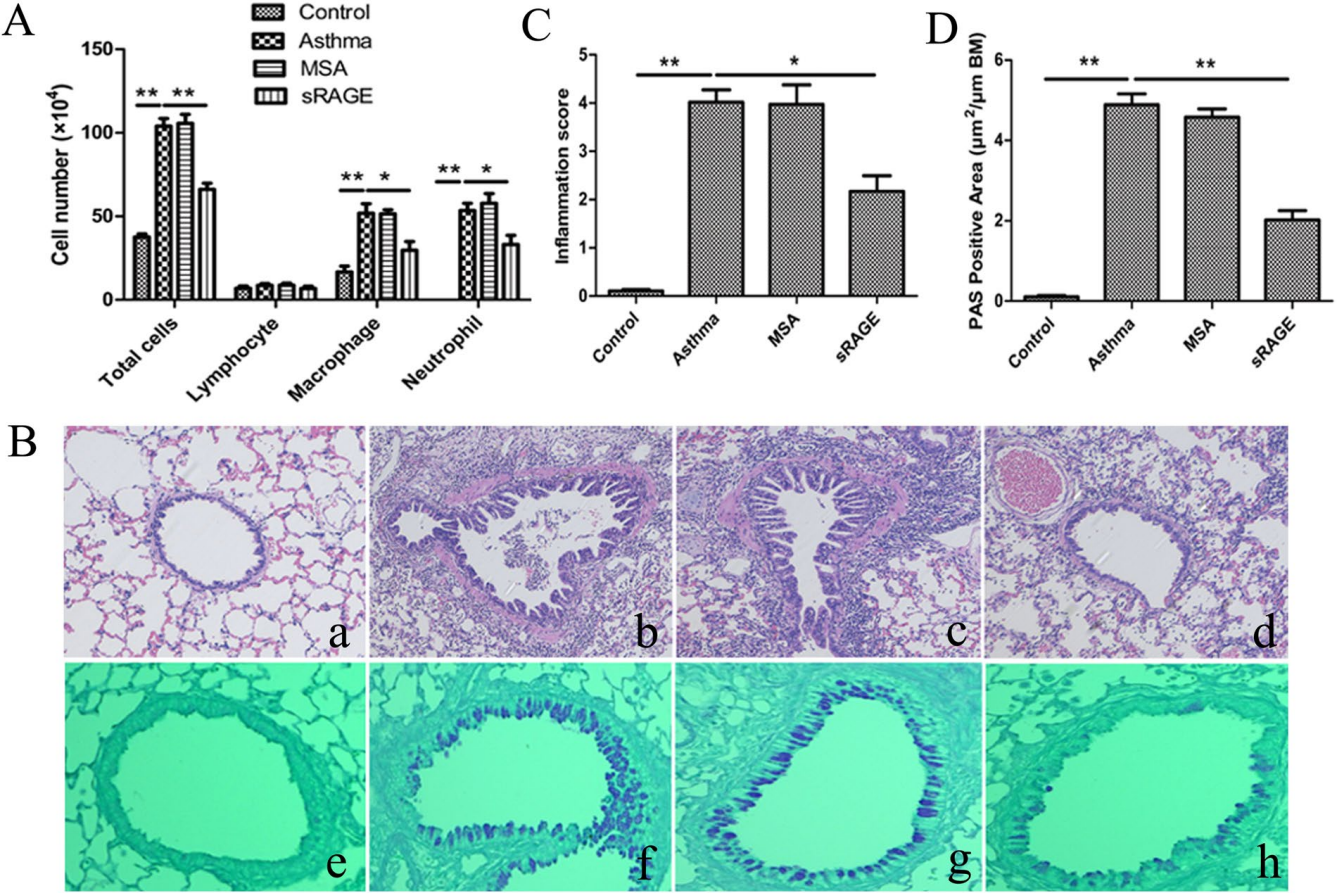
Local administration of sRAGE inhibits neutrophilic airway inflammation. (**A**) sRAGE inhibited the infiltration of neutrophils and macrophages into BALF. BALF was collected at 24 h after the last OVA challenge. The numbers of total and differential cell counts in the BALF of mice in Control, Asthma, MSA-treated, and sRAGE-treated groups were analysed by counting. Values represent the means ± SEM (n = 5–6). *P < 0.05 and **P < 0.01 compared with the Asthma group. (**B**) Representative H&E and PAS staining of lung tissues. H&E-stained lung tissues (upper panel) from mice in Control (a), Asthma (b), MSA-treated (c), and sRAGE-treated groups (d). PAS-stained lung tissues (lower panel) from mice in Control (e), Asthma (f), MSA-treated (g), and sRAGE-treated groups (h). (**C**) Inflammation scores were decreased in sRAGE-treated mice. Values represent the means ± SEM (n = 5–6). *P < 0 05 and **P < 0 01 compared with the Asthma group. (**D**) The proportion of PAS-positive areas along the airways area was decreased in sRAGE-treated mice. Values represent the means ± SEM (n = 5–6). **P < 0 01 compared with the Asthma group.

Because the decreased expression of sRAGE in asthmatic mice was closely related to neutrophil infiltration and IL-17 production, we next determined whether administration of sRAGE decreased neutrophilic inflammation and Th17 cytokine production in asthmatic mice. To evaluate the effects of sRAGE in allergen-primed airway inflammation, we locally administrated sRAGE or MSA intranasally before OVA plus LPS sensitisation. As expected, there was a substantial increase in total bronchoalveolar (BAL) cells and neutrophils in OVA plus LPS-sensitised mice upon subsequent OVA challenge, but almost no eosinophils, which is one of the characteristics of neutrophilic bronchial asthma. However, pretreatment with sRAGE led to a significant decrease in the number of total BAL cells as well as neutrophils and macrophages in comparison with MSA-treated mice (Fig. 4A).

To further characterise the effects of sRAGE on OVA-induced airway inflammation, we compared lung histology in various groups of mice. As shown in haematoxylin and eosin (H&E)-stained sections (Fig. 4B), OVA plus LPS-sensitised and OVA challenged mice showed increased numbers of inflammatory cells in peribronchial and perivascular regions, which were rarely detected in PBS-sensitised mice. In contrast, sRAGE administration significantly alleviated airway inflammation because we observed little peribronchiolar and perivascular infiltrates in the airways (Fig. 4B). Histological examination also showed decreased periodic acid-Schiff (PAS)-positive mucus-containing goblet cells in sRAGE-treated mice compared with those in MSA-treated mice (Fig. 4B). Collectively, local administration of sRAGE before sensitisation to OVA plus LPS in the mouse model of neutrophilic asthma substantially lessened the severity of neutrophilic airway inflammation.

We also evaluated whether administration of sRAGE before allergen challenge decreased neutrophilic inflammation in asthmatic mice. Sensitised animals treated with sRAGE before allergen challenge exhibited no effects on the appearance of inflammatory cell infiltration in lung tissue (Supplementary Fig. S2). These data provide evidence for the beneficial role of sRAGE in the early phase of neutrophilic asthma.

### sRAGE inhibits the development of airway hyperreactivity (AHR)

**Figure 5:**
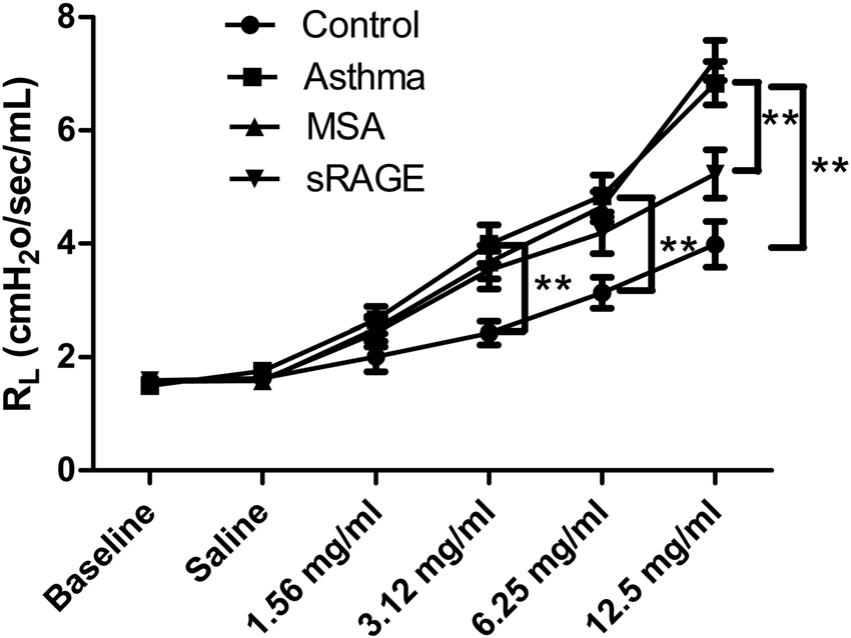
Local administration of sRAGE decreases AHR. Changes in lung resistance (R_L_) in response to increasing doses of methacholine represents OVA-induced AHR and were measured at 48 h after the final challenge in mice from Control, Asthma, MSA-treated, and sRAGE-treated groups. Values represent the means ± SEM (n = 5–6). **P < 0.01 compared with the Asthma group.

To assess the effect of sRAGE on AHR, we evaluated airway responsiveness to methacholine (MCh) inhalation. After OVA challenge, AHR was significantly higher in OVA plus LPS-sensitised mice upon subsequent OVA challenge. As expected, administration of sRAGE before airway sensitisation significantly inhibited the development of AHR (Fig. 5).

### sRAGE suppresses local Th17 skewing *in vivo*

**Figure 6:**
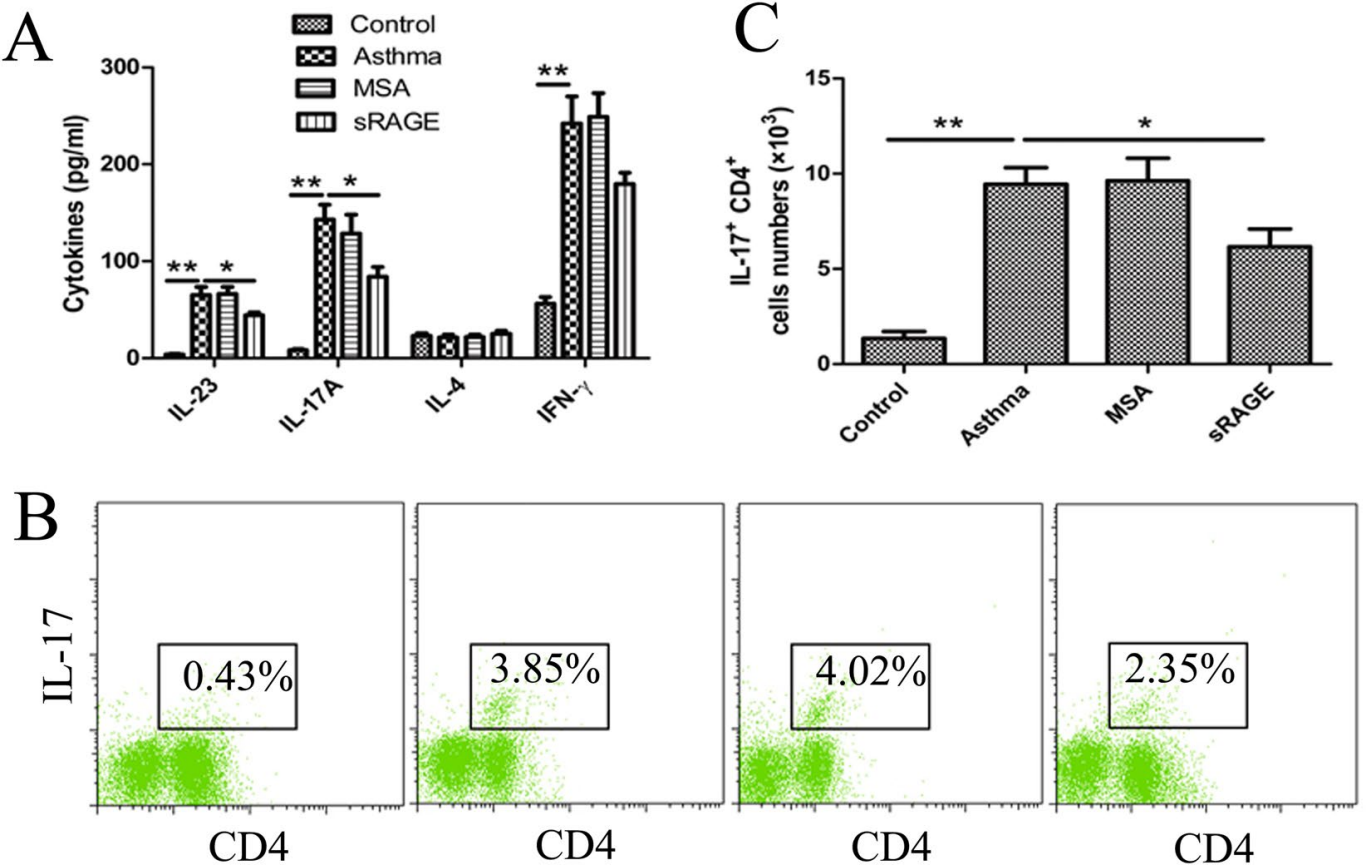
Local administration of sRAGE suppresses the Th17 response. (**A**) sRAGE decreased Th17 cytokine production after OVA challenge. BALFs were collected at 24 h after the last OVA challenge. The levels of IL-23, IL-17A, IL-4, and IFN-γ in the BALF of mice from Control, Asthma, MSA-treated, and sRAGE-treated groups were assessed by ELISAs. Values represent the means ± SEM (n = 5). *P < 0.05 and **P < 0.01 compared with the Asthma group. (**B**) sRAGE down-regulated the count of Th17 cells in the lung. Representative results of the percentage of Th17 cells in CD3^+^-gated lung cells from mice in Control (a), Asthma (b), MSA-treated (c), and sRAGE-treated groups (d) were analysed by flow cytometry. (**C**) Histogram showing the absolute counts of Th17 cells among the lung cells of mice from the various groups. Values represent the means ± SEM (n = 5). *P < 0.05 and **P < 0.01 compared with the Asthma group.

To analyse the immune response in airways, cytokine levels were measured in BALF. As shown in Fig. 6A, local administration of sRAGE resulted in a small reduction in the levels of IL-17 and IL-23 (Th17-associated cytokines). In contrast, there were no significant differences in the levels of IL-4 (a Th2-associated cytokine) or interferon (IFN)-γ (a Th1-associated cytokine) compared with mice that received MSA.

Because administration of sRAGE inhibited the IL-17A level in BALF, we investigated the possibility that this inhibition was mediated through the decreased production of Th17 cells in the lung. As shown in Fig. 6B, OVA plus LPS-sensitised mice exhibited a dramatic increase of Th17 cell production in their lung tissue, whereas administration of sRAGE significantly down-regulated the production of Th17 cells in lung tissue. These results suggested that local administration of sRAGE suppresses Th17 skewing in the mouse model of neutrophilic asthma.

### sRAGE decreases RAGE and IL-23 expression in CD11c^+^ APCs of lung tissue

**Figure 7:**
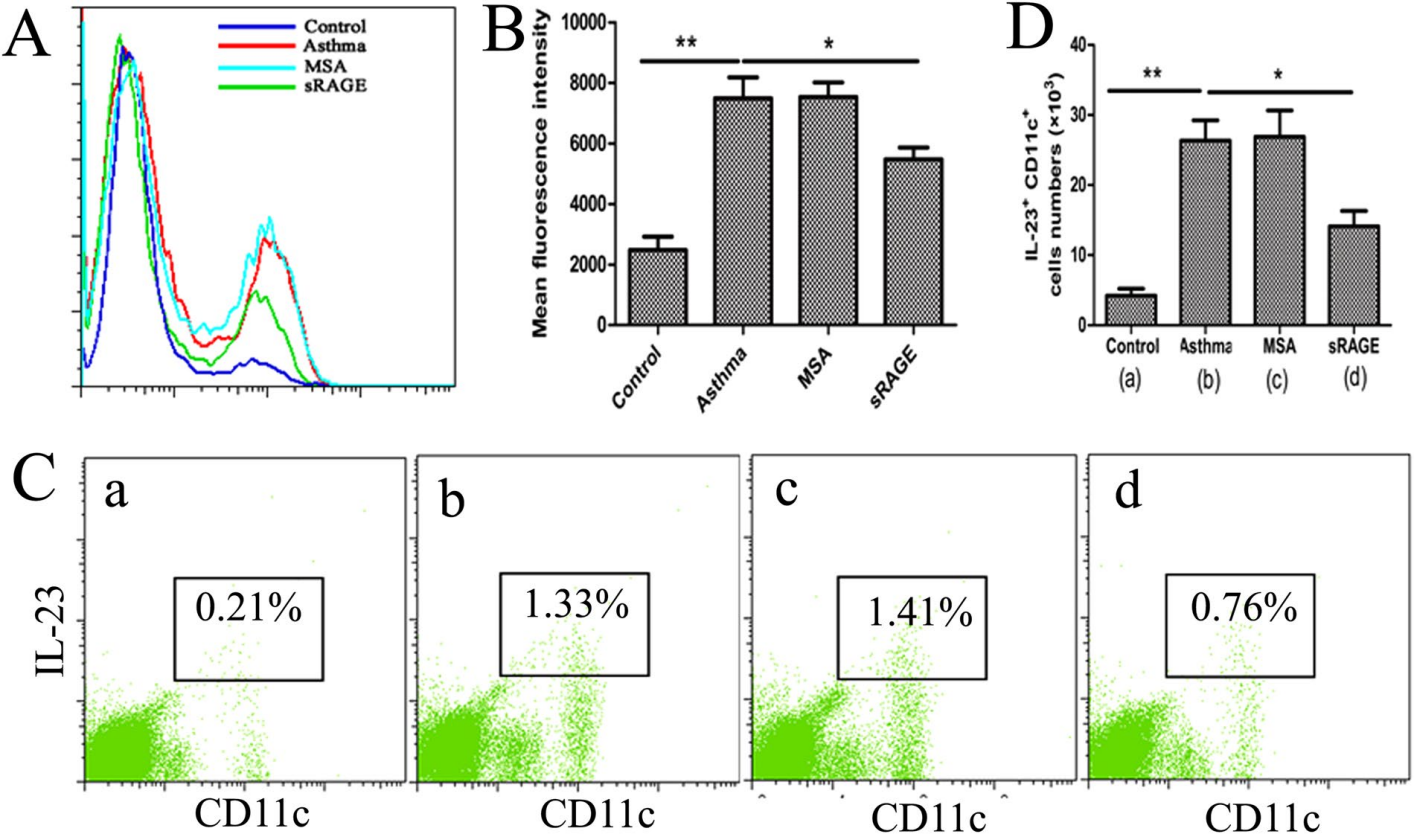
Local administration of sRAGE inhibits RAGE and IL-23 expression by lung CD11c^+^ APCs. (**A**) sRAGE inhibited RAGE expression in CD11c^+^ APCs. Representative findings of RAGE expression in CD11c^+^ APCs of mice from Control, Asthma, MSA-treated, and sRAGE-treated groups were analysed by flow cytometry. (**B**) Mean fluorescence intensity (MFI) showing the expression of RAGE in CD11c^+^ APCs of mice from the various groups. Values represent the means ± SEM (n = 5). *P < 0.05 and **P < 0.01 compared with the Asthma group. (**C**) sRAGE inhibited the percentage of IL-23^+^ CD11c^+^ APCs among low density lung cells. Representative results of the percentage of IL-23^+^ CD11c^+^ APCs in mice from Control (a), Asthma (b), MSA-treated (c), and sRAGE-treated groups (d) were analysed by flow cytometry. (**D**) Histogram showing the absolute counts of IL-23^+^ CD11c^+^ APCs in mice from the various groups. Values represent the means ± SEM (n = 5). *P < 0.05 and **P < 0.01 compared with the Asthma group.

Because DCs possess the potential to induce Th cell polarisation and play a vital role in sensitisation to the development of asthma, we investigated whether pulmonary DCs are involved in the anti-inflammatory effects of sRAGE in Th17 polarisation. We first assessed RAGE expression in lung DCs, a prerequisite for initiating the immune response to AGE signalling in airways. As shown in Fig. 7A, pretreatment with sRAGE induced a remarkable reduction in RAGE expression by pulmonary DCs in terms of mean fluorescence intensity (MFI) compared with MSA-treated mice (Fig. 7B), suggesting that RAGE expression in lung DCs was substantially affected by the inhibition of HMGB1 signalling with sRAGE.

To eliminate the possibility of sRAGE masking the RAGE antibody epitope, sRAGE was directly added to lung cell suspensions from asthmatic mice, and then RAGE expression in DCs was evaluated using the RAGE antibody for flow cytometric analysis. The results showed that administration of sRAGE had no effect on RAGE expression in DCs (Supplementary Fig. S2), suggesting that sRAGE did not mask the RAGE antibody epitope.

Additionally, we further assessed the polarising ability of DCs in generating Th17 cells. Consistent with the reduced level of IL-23 in BALF, administration of sRAGE significantly decreased IL-23 production by lung CD11c^+^ APCs, which is an important Th17-polarised cytokine that promotes the differentiation and proliferation of Th17 cells (Fig. 7C,D). These results demonstrated that sRAGE decreased the number of IL-23^+^ CD11c^+^ APCs, thus inhibiting development of the Th17-mediated immune response in the lungs.

### sRAGE inhibits recombinant HMGB1 (rHMGB1)-activated bone marrow-derived DCs (BMDCs) inducing Th17 responses *in vitro*

**Figure 8:**
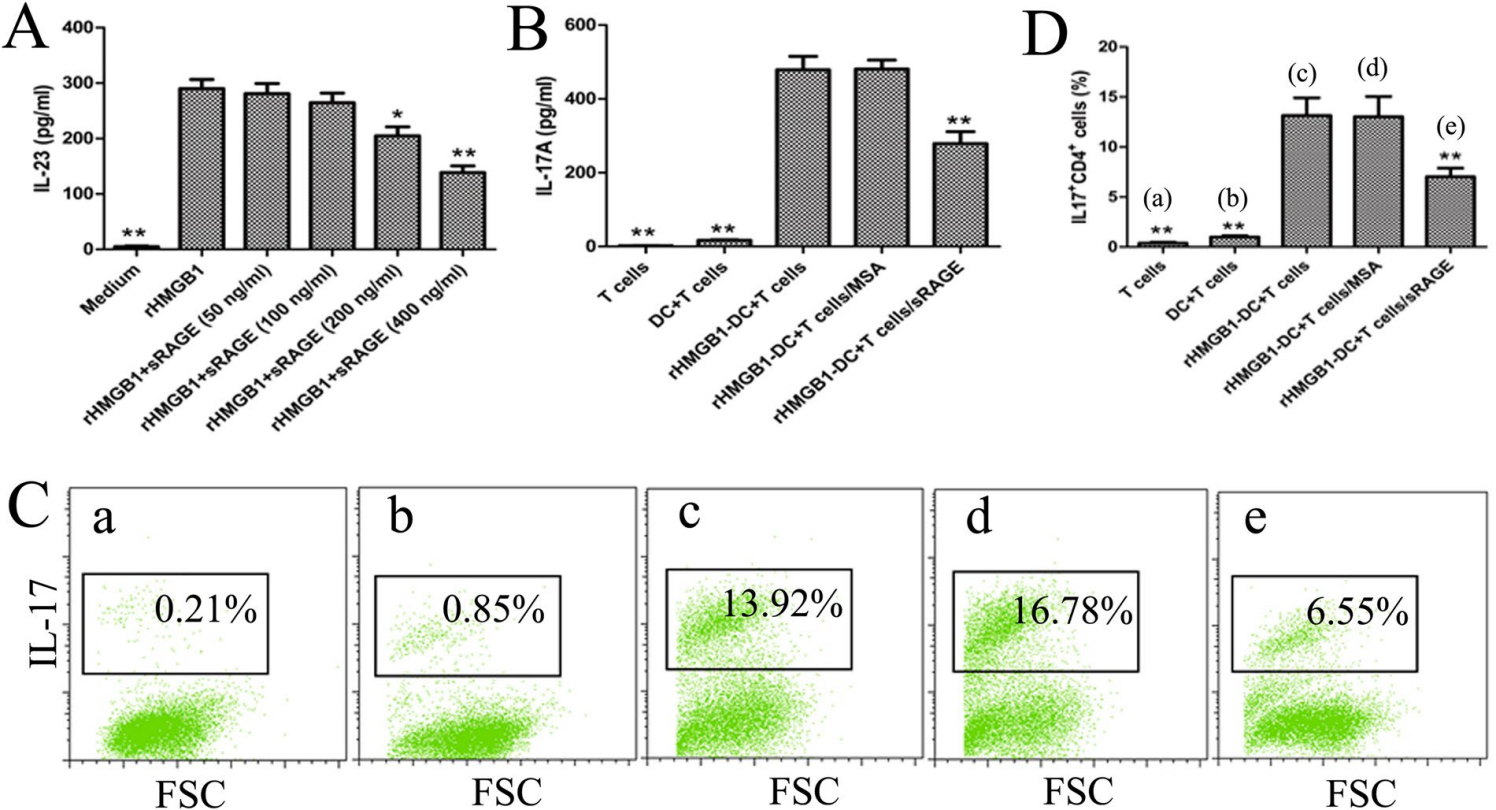
sRAGE inhibits rHMGB1-stimulated BMDCs inducing Th17 responses *in vitro*. (**A**) sRAGE inhibited rHMGB1-stimulated BMDCs to produce IL-23. BMDCs were cultured in medium or stimulated with 500 ng/ml HMGB1 alone or cocultured with graded concentrations of sRAGE (50, 100, 200, 400 ng/ml) for 2 days. IL-23 levels in culture supernatants were measured using an ELISA. Values represent the means ± SEM (n = 5). *P < 0.05 and **P < 0.01 compared with the rHMGB1-stimulated BMDC group. (B, C, and D) sRAGE inhibited Th17 polarisation induced by rHMGB1-stimulated BMDCs. CD4^+^ T cells from the spleens of OVA-sensitised mice (1 × 10^5^) were cultured alone or cocultured with BMDCs (2.5 × 10^4^) that were treated with or without 500 ng/ml rHMGB1 in the presence or absence of 400 ng/ml sRAGE or MSA. The culture supernatants were analysed for IL-17A using an ELISA (**B**), and the percentages of IL-17^+^ CD4^+^ T cells were analysed by flow cytometry (**C**). (**D**) Histogram showing the percentages of IL-17^+^ CD4^+^ T cells after the 5 days of coculture. Values represent the means ± SEM (n = 5). *P < 0.05 and **P < 0.01 compared with the rHMGB1-stimulated BMDC group.

Our previous study showed that rHMGB1-activated BMDCs have the potential to induce efficient Th17 polarisation via endogenous production of IL-23^19^. Considering the role of HMGB1 as the key instigator of adaptive immune responses for BMDC-mediated Th17 polarisation, blockade of HMGB1 signalling might represent a major mechanism of sRAGE-mediated immune regulatory effects. To examine the contribution of sRAGE to Th17 cell development induced by rHMGB1-activated BMDCs, Th17 polarisation *in vitro* was analysed by co-culture of OVA-pulsed BMDCs and CD4^+^ T cells from OVA-sensitised mice in the absence or presence of rHMGB1 and/or sRAGE. As a result, rHMGB1-activated BMDCs significantly enhanced secreted IL-17A levels in the culture supernatants (Fig. 8A) and increased the percentage of IL-17^+^ CD4^+^ T cells (Fig. 8B). In contrast, high concentrations of sRAGE (200 or 400 ng/ml) significantly down-regulated the expression of IL-17 induced by rHMGB1-stimulated BMDCs, suggesting that sRAGE suppressed the Th17 response induced by rHMGB1-stimulated BMDCs.

### sRAGE inhibits the Th17 priming induced by HMGB1-activated BMDCs *in vivo*

**Figure 9:**
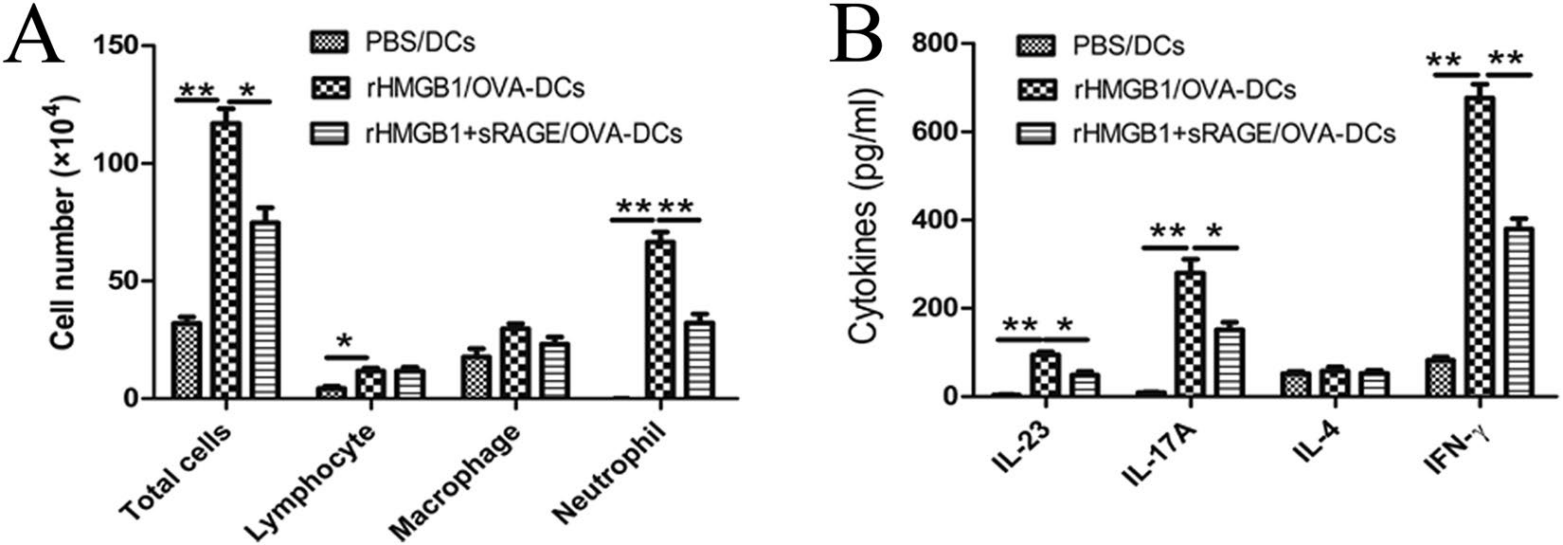
sRAGE impedes the potential of rHMGB1-stimulated BMDCs to prime Th17 responses *in vivo*. Mice received an intratracheal (i.t.) injection of 2 × 10^6^ PBS-treated, non-pulsed DCs (PBS/DCs), rHMGB1-treated, OVA-pulsed DCs (rHMGB1/OVA-DCs), or rHMGB1 plus sRAGE-treated, OVA-pulsed DCs (rHMGB1 + sRAGE/OVA-DCs) and were then exposed to OVA aerosols on days 10–12. One day after the final challenge, the mice were sacrificed for further analysis. (**A**) Total and differential cells counts and (**B**) cytokine profiles were analysed in the BALF. Values represent the means ± SEM (n = 5). *P < 0.05 and **P < 0.01 compared with the Asthma group.

To evaluate the role of sRAGE in HMGB1-activated BMDCs of Th17 polarisation *in vivo*, we adoptively transferred rHMGB1 or rHMGB1 plus sRAGE-treated DCs into the airways of mice (Fig. 1B). Adoptive transfer of HMGB1-activated DCs resulted in significant neutrophilic inflammation characterised by infiltration of neutrophils into the BALF (Fig. 9A) and Th17/Th1 priming as shown by increases in IL-23, IL-17A, and IFN-γ levels of the BALF (Fig. 9B). However, adoptive transfer of HMGB1 plus sRAGE-treated DCs markedly decreased the neutrophils in the BALF (Fig. 9A) and the Th17 response as shown by decreases in IL-23 and IL-17A levels (Fig. 9B). These data suggested that sRAGE inhibits the capability of HMGB1-activated DCs to induce neutrophilic inflammation and Th17 skewing in asthmatic mice.

## Discussion

Neutrophilic asthma is a prevalent, yet recently described phenotype of asthma, characterised by the mass infiltration of neutrophilic rather than eosinophilic airway inflammation and AHR, which may have an infectious origin^27^. Th17 immune responses may have a role in the pathogenesis of neutrophilic asthma. However, the nature of the association between Th17 responses and the development of neutrophilic asthma is not understood. DCs have been regarded as critical initiators of allergic inflammation, not only because of their abilities to process and present inhaled antigens, but also their capabilities to activate naïve T cells and even determine the type of immune response^28^. Some experiments indicate that HMGB1 is involved in local inflammatory responses and contributes to direct DC functions^29,30^. Therefore, we postulated that HMGB1 acts on DCs to promote the local Th17 response in neutrophilic asthma.

Previous studies have revealed up-regulation of HMGB1 expression in a variety of diseases, and activation of HMGB1/RAGE signalling is a poor prognostic factor for these diseases. For example, HMGB1/RAGE signalling is upregulated in patients with diabetes and atherosclerosis^31^. Some investigations have reported that reduced sRAGE is associated with neutrophilic airway inflammation in asthma and chronic obstructive pulmonary disease (COPD)^25,32^, suggesting that sRAGE might have a protective role in inflammatory lung diseases. Here, we confirmed that production of sRAGE was significantly decreased in the mouse model of neutrophilic asthma, and this decrease was well correlated with elevated IL-17 levels and increased neutrophil infiltration, suggesting that sRAGE production was responsible for the development of Th17-biased inflammation. Furthermore, our study showed that administration of sRAGE inhibited HMGB1 expression, airway neutrophilic inflammation, and AHR in the mouse model of neutrophilic asthma, indicating that HMGB1/RAGE signalling is a key regulator of the immune cascade leading to subsequent Th17 polarisation, neutrophilic airway inflammation, and AHR.

RAGE is a pattern recognition receptor that binds AGEs, HMGB1, amyloid β-peptide, and S100 protein. It interacts with diverse endogenous ligands and elicits activation of immune and inflammatory responses, induction of oxidant stress, and tissue remodelling responses^33^. Recently, several carboxyl-terminal truncated isoforms of RAGE, such as sRAGE and endogenous secretory RAGE (esRAGE), were identified in the lungs of both humans and mice^34,35^. Because these isoforms lack a transmembrane domain, they are secreted and act as decoy receptors. Indeed, sRAGE has been shown to prevent or reverse RAGE signals in experimental models of diabetic atherosclerosis, wound healing, amyloidosis, and colitis^36–39^. In addition, Zhang and colleagues reported that sRAGE treatment significantly attenuates the neutrophilic inflammation and pathological changes in the LPS-induced murine model of acute lung injury, suggesting that sRAGE may be secreted as a decoy receptor and contributes to the suppression of excessive inflammatory responses during actual lung injury^40^. Izushi and colleagues reported that the release of sRAGE has the potential to attenuate the excessive inflammatory response in acute respiratory distress syndrome (ARDS), and supports the notion that RAGE may be a promising candidate as a molecular target for the treatment of ARDS^41^. Sukkar and colleagues reported that systemic sRAGE was significantly decreased in subjects with neutrophilic asthma or COPD compared with those without airway neutrophilia, and that neutrophilic airway inflammation in asthma and COPD is associated with reduced sRAGE. They proposed that sRAGE deficiency is a primary consequence of excessive neutrophilic inflammation. They also identified sRAGE as a potential biomarker for the prognosis or management of asthmatic or COPD subjects with neutrophilic airway inflammation^25^. Smith and colleagues reported lower plasma levels of sRAGE in COPD patients during disease exacerbation, which is frequently caused by respiratory bacterial and/or viral infection, compared with patients with stable COPD. These data support the theory that sRAGE sequesters RAGE ligands and limits RAGE signalling, and that correcting deficiencies in sRAGE could represent a therapeutic strategy for neutrophilic asthma and COPD^42^. Milutinovic and colleagues reported that the absence of RAGE abolishes most assessed measures of pathology, including airway hypersensitivity, eosinophilic inflammation, and airway remodelling in a house dust mite mouse model of asthma^43^. All these studies indicate that RAGE inhibition or blockage with sRAGE may serve as a promising therapeutic strategy to control airway inflammation. However, its protective effects and the molecular mechanisms of sRAGE in neutrophilic asthma have not been characterised previously.

IL-17 has been suggested to play a role in Th17 responses. In this study, IL-17 was up-regulated in the BALF of asthmatic mice, and administration of sRAGE actively inhibited the polarisation of Th17 cells and expression of IL-23 in CD11^+^ APCs. These results suggest that sRAGE is a negative regulator of established allergic asthma and may drive the IL-23/IL-17 axis that in turn controls the Th17 response in neutrophilic asthma. More interestingly, RAGE expression in pulmonary DCs was regulated by sRAGE treatment, implying that negative feedback through HMGB1 signalling might exist to inhibit the development of allergic inflammation.

HMGB1/RAGE signalling not only occurs in DCs but also in other inflammatory cells^44^. To rule out the direct effects of HMGB1 blockage by sRAGE on other effector cells in allergic inflammation, we performed experiments in which rHMGB1-activated DCs were treated with sRAGE *in vitro* prior to adoptive transfer into the airways of naive mice. As expected, rHMGB1-activated DCs triggered strong neutrophilic airway inflammation that was accompanied by massive production of Th17 cytokines such as IL-23 and IL-17. However, pretreatment of HMGB1-activated DCs with sRAGE resulted in a significant reduction of neutrophil infiltration as well as IL-23 and IL-17 levels, further illuminating the putative role of HMGB1 signalling in DC-primed allergic diseases and demonstrating the potential of HMGB1 signalling blockade in DCs to control Th17 responses and neutrophilic airway inflammation.

In conclusion, our findings indicate that local administration of sRAGE inhibits Th17-mediated cardinal features of neutrophilic asthma at least partly by blocking HMGB1 signalling in lung DCs. Therefore, it might be worth determining whether decreased levels of sRAGE correlate with the severity of asthma, and further studies on the physiological and pathological significance of sRAGE may shed more light on allergic pathogenesis and reveal a new therapeutic perspective for these diseases.

## Methods

### Mice

Female 8–10-week-old C57BL/6 mice were obtained from the Animal Center of Jinling Hospital. All mice were bred and maintained under pathogen-free conditions, where the temperature was maintained at 20–22 °C and the humidity was kept at 50–60%. The dark/light cycles were 12 h each. All experiments involving animals and tissue samples were performed according to the guidelines of the National Institutes of Health and Medical School of Nanjing University (Nanjing, China), and all procedures were approved by the Institutional Animal Care and Use Committee of Medical School of Nanjing University.

### Protocols for the mouse model and experimental intervention

A murine model of neutrophilic asthma characterised by Th17 cell responses was generated as described previously^45^. Briefly, mice underwent intranasal sensitisation with 75 *μ*g OVA (grade V; Sigma-Aldrich, St Louis, MO, USA) plus 10 *μ*g LPS (*E. coli* serotype 026:B6; Sigma-Aldrich) on days 0, 1, 2, and 7, and were then challenged by intranasal instillation of 50 *μ*g OVA alone on days 14, 15, 21, and 22. All mice were analysed at 24 h after the last OVA challenge.

In this study, the mice were divided randomly into four groups (n = 5–6 mice each) as follows: (i) mice sensitised with PBS and challenged with OVA (Control group); (ii) mice sensitised with OVA plus LPS and challenged with OVA (Asthma group); (iii) mice treated with MSA (Sigma-Aldrich) at 30 min before sensitisation to OVA plus LPS and the same challenge with OVA (MSA group); (iv) mice treated with sRAGE (R&D Systems) at 30 min before sensitisation to OVA plus LPS and the same challenge with OVA (sRAGE group). MSA or sRAGE was administered intranasally (200 *μ*g/kg) on days 0, 1, 2, and 7 before sensitisation (Fig. 1A). The dose of sRAGE was predetermined by staining analysis of airway inflammation in mice that received 100–400 *μ*g/kg sRAGE. In some experiments, different dosages of sRAGE (200 or 400 *μ*g/kg) were administered intranasally to the mice at 30 min before the secondary OVA challenges on days 14, 15, 21, and 22. One the day after the final challenge, the mice were sacrificed for histological analysis.

### BALF

To assess differential BALF cell counts, lungs were lavaged three times with 0.75 ml Ca^2+^ and Mg^2+^-free Hank’s balanced salt solution containing 0.1 mM sodium EDTA. Cells in the BALF samples were centrifuged at 300 *g* for 5 min to generate a cell pellet and cell-free supernatant that was frozen and stored at −80 °C until analysis by enzyme-linked immunosorbent assays (ELISAs). For differential cell counts, cytocentrifuged preparations were fixed and stained with Diff-Quick (Kokusaishiyaku, Kobe, Japan) and differentiated morphologically by counting 300 cells/slide. The levels of sRAGE, IL-23, IL-17A, IL-4, and IFN-γ in the BALF were determined by ELISAs according to the manufacturer’s instructions (eBioscience, CA, USA).

### Histological examination

For histopathological assessment, non-lavaged lobes of the lungs were fixed and embedded in paraffin. Sections (5 *µ*m thick) from all lobes were prepared and stained with H&E for analysis of cellular infiltrates. Mucus-containing goblet cells were detected by staining with PAS. The numbers of PAS-positive goblet cells were determined only in cross-sectional areas of the airway wall. The sections were observed under a microscope at × 200 magnification. Six to eight fields per slide in five to six samples from each group of mice were examined in a blinded manner.

### AHR

Airway responsiveness of mice to increasing concentrations of aerosolised MCh was measured as described in detail previously^46^. After the mice were anesthetised with an intraperitoneal injection of pentobarbital sodium (100 mg/kg), the trachea was cannulated via tracheotomy. The mice were connected to a computer-controlled, small animal ventilator and ventilated with a tidal volume of 10 ml/kg at a frequency of 150 breaths per min using the flexiVent System (SCIREQ, Montreal, Canada). After obtaining baseline measurements, each mouse was challenged with MCh aerosol at increasing concentrations (0, 1.56, 3.12, 6.25, and 12.5 mg/ml), and the lung resistance (R_L_) to the inhaled MCh was recorded.

### Measurement of HMGB1 expression

Immunohistochemical staining of lung tissue was performed to detect the expression of HMGB1 in the lungs as described previously^47^. In brief, 5 *μ*m paraffin-embedded sections were stained with a primary antibody against HMGB1 (Santa Cruz Biotechnology Inc., CA, USA), which was diluted at 1:50, and a biotinylated secondary antibody that was diluted at 1:500. The bound peroxidase was visualised using the 3,3′-diaminobenzidine method. Ten randomly chosen fields per section were counted under a microscope at × 200 magnification, and the intensity of HMGB1 protein staining was determined as the average optical density using IPP software (Image-Pro Plus 6.0, Media, Cybernetics).

HMGB1 expression was also analysed by western blotting. Lung tissues were homogenised to prepare lung lysates. Protein samples (30 *μ*g) were subjected to 10% SDS-polyacrylamide gel electrophoresis and then transferred to nitrocellulose membranes. The membranes were blocked in blocking buffer (5% dry milk powder in TBS plus Tween 20) for 1 h at room temperature. Then, the membranes were incubated with a primary HMGB1 antibody (Santa Cruz Biotechnology Inc.). In some experiments, 200 or 400 ng/ml sRAGE was directly added to the lung homogenates, and HMGB1 expression was evaluated using the HMGB1 antibody by western blotting. HMGB1 mRNA levels were analysed in the lung tissue by quantitative PCR as described previously^48^.

### Culture and treatment of DCs

BMDCs were isolated and cultured in DC medium as described previously^49^. After 8 days of culture, DCs were enriched using anti-CD11c-coated magnetic microbeads (Miltenyi-Biotec, Auburn, CA, USA) and treated with medium in the absence or presence of rHMGB1 (500 ng/ml) (R&D Systems, Minneapolis, MN, USA) with or without various concentrations of sRAGE (10, 100, 200, or 400 ng/ml) (R&D Systems) for 2 days. Cytokine concentrations in the cell culture supernatants were measured using an IL-23 ELISA kit (R&D Systems). rHMGB1 and sRAGE were tested by Limulus amebocyte lysate (ZhanJiang A&C Biological, China) and considered to be endotoxin free. In addition, rHMGB1 was in the disulphide form in this study.

### Coculture of DCs with CD4^+^ T Cells

CD4^+^ T cells were highly enriched from the spleens of OVA-sensitised mice using a Mouse CD4^+^ T cell enrichment kit (Stem Cell Technologies, Vancouver, Canada) following the manufacturer’s instructions^50^. The OVA-sensitised mouse model was established as described previously^30^. The purity of isolated CD4^+^ T cells was greater than 98%. CD4^+^ T cells (1 × 10^4^ cells/well) were mixed in a 96-well plate with BMDCs (2.5 × 10^4^/well) stimulated with rHMGB1 (500 ng/ml) in the absence or presence of sRAGE (200 ng/ml). OVA (10 *μ*g/mL) was added, and the mixed cells were cultured at 37 °C with 5% CO_2_. After 5 days of incubation, the culture supernatants were analysed for IL-17A by an ELISA, and the expression of intracellular IL-17 in CD4^+^ T cells was analysed by flow cytometry.

### Flow cytometric analysis

To detect IL-17^+^ CD4^+^ T cells in the DC-T cell coculture system, the cells were incubated with 10 *μ*g/ml brefeldin A (eBioscience) for 2 h and then stained for cell surface CD4 by a FITC-anti-CD4 mAb (eBioscience) for 30 min at 4 °C. After incubation in a fixation/permeabilisation solution (eBioscience), the cells were stained for intracellular IL-17 with a PE-anti-IL-17 mAb (eBioscience) for 30 min and analysed using a FACSCalibur flow cytometer (BD Biosciences).

To detect IL-17^+^ CD4^+^ T cells in lung tissue, lung cells were obtained according to previously reported methods^19^. Lung cells (4 × 10^6^/ml) were washed three times in FACS buffer (PBS containing 1% bovine serum albumin and 0.1% sodium azide), incubated with brefeldin A (10 *μ*g/ml) for 2 h, and then stained with surface-specific Abs (anti-CD3-APC and anti-CD4-FITC; eBioscience) for 30 min at 4 °C. For intracellular staining, cells were fixed and permeabilised with the fixation/permeabilisation solution, intracellularly stained with the PE-anti-IL-17 mAb for 30 min, and subsequently analysed using the FACSCalibur flow cytometer.

To detect RAGE and IL-23 expression in CD11C^+^ APCs, low density lung cells were enriched as described previously^19^. Briefly, digested lung cells (10 × 10^6^/ml) were filtered through a nylon wool plug and resuspended in high density Percoll (ρ = 1.075 g/ml), overlaid with an equal volume of lower density Percoll (ρ = 1.030 g/ml), and centrifuged at 400 × *g* for 20 min. Low density lung cells, which were enriched for mononuclear cells, were recovered from the 1.075/1.030 Percoll interface and washed with Hank’s balanced salt solution. Low density lung cells were then stained with a DC-specific Ab (FITC-anti-CD11C mAb, eBioscience) and PE-conjugated goat anti-mouse RAGE mAb (Sigma-Aldrich) for 30 min at 4 °C. For intracellular staining, cells were fixed and permeabilised with the fixation/permeabilisation solution, stained with PE-anti-IL-23 mAb (eBioscience) for 30 min, and subsequently analysed using the FACSCalibur flow cytometer. In some experiments, 200 or 400 ng/ml sRAGE was directly added to lung cell suspensions from asthmatic mice, and RAGE expression in DCs was evaluated using the RAGE antibody by flow cytometry.

### Adoptive transfer of DCs

A murine model of asthma through the transfer of BMDCs was established as described previously^51^. Briefly, BMDCs were enriched and pulsed with OVA overnight (OVA-DCs). OVA-DCs were then injected into naïve mice via the intratracheal route (i.t.). Ten days after intratracheal immunisation, the mice were challenged with OVA (1% w/v in PBS; grade V; Sigma-Aldrich) aerosol during a daily, 30 min challenge on 3 consecutive days. In this experiment, the mice were divided randomly into the three groups (n = 5–6 per group) as follows: (i) mice received an i.t. injection of 2 × 10^6^ PBS-treated and non-pulsed DCs (PBS/DCs); (ii) mice received an i.t. injection of 2 × 10^6^ rHMGB1-treated OVA-DCs (rHMGB1/OVA-DCs); (iii) mice received an i.t. injection of 2 × 10^6^ rHMGB1 plus sRAGE-treated OVA-DCs (rHMGB1 + sRAGE/OVA-DCs). The OVA-DCs were treated with rHMGB1, sRAGE, or control IgG at concentrations of 500, 200, or 200 ng/ml, respectively. All mice were sacrificed at 24 h after the final challenge for further analyses.

### Statistical analysis

Data are expressed as means ± standard error of the mean (SEM). Differences between groups were analysed by SPSS for windows (version 16.0) using the unpaired, two-tailed, parametric Student t-test or one-way analysis of variance followed by Dunnett’s multiple comparison tests. *P*-values of less than 0.05 were considered as statistically significant.

## Electronic supplementary material

Supplementary information
