# Supplementary material for: sRAGE alleviates neutrophilic asthma by blocking HMGB1/RAGE signalling in airway dendritic cells
Source: Sci Rep. 2017 Oct 27;7:14268. doi: 10.1038/s41598-017-14667-4 (PMC5660212; doi:10.1038/s41598-017-14667-4)

Supplementary Information

sRAGE alleviates neutrophilic asthma by blocking HMGB1/RAGE signalling in airway dendritic cells

Fang Zhang[1], Xin Su[1], Gang Huang[2], Xiao-Feng Xin[1], E-Hong Cao[1], Yi Shi[1], Yong Song[1]

[1]Department of Pulmonary Medicine, Jinling Hospital, Nanjing University School of Medicine, Nanjing, China

[2]Department of Medical Genetics, The Third Military Medical University, Chongqing, China

A

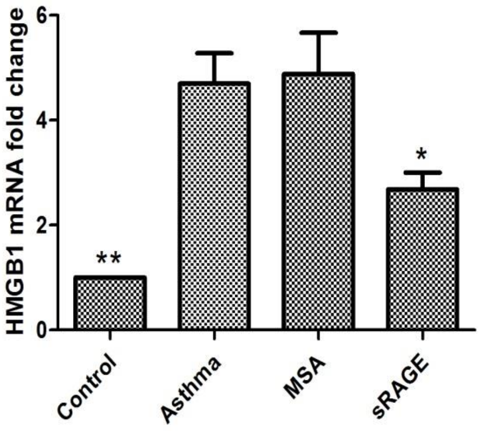
HMGB1 mRNA fold change
0
2
4
6
**
*
Control
Asthma
MSA
sRAGE

B

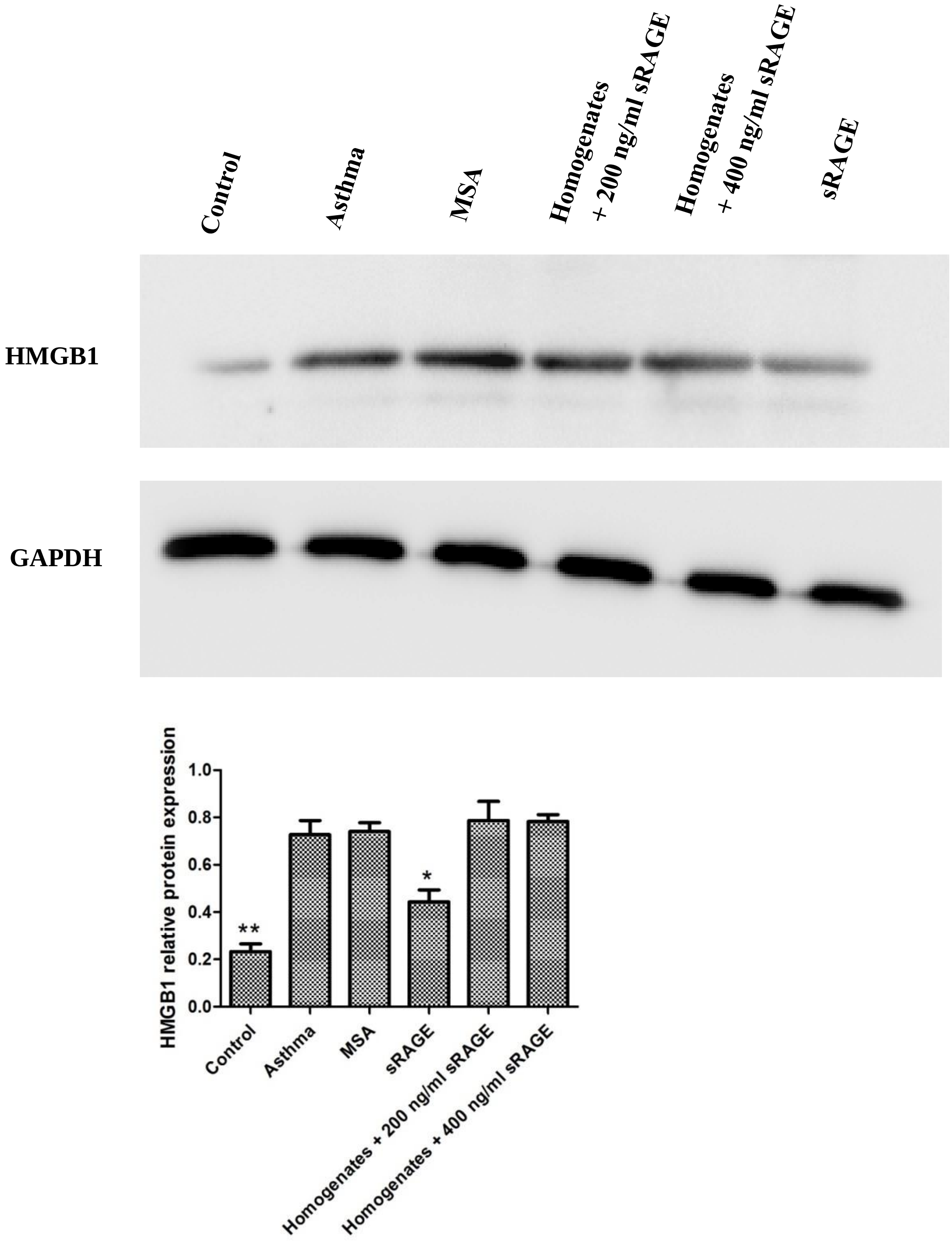
Control
Asthma
MSA
Homogenates + 200 ng/ml sRAGE
Homogenates + 400 ng/ml sRAGE
sRAGE
HMGB1
GAPDH
HMGB1 relative protein expression
0.0
0.2
0.4
0.6
0.8
1.0
**
*
Control
Asthma
MSA
sRAGE
Homogenates + 200 ng/ml sRAGE
Homogenates + 400 ng/ml sRAGE

Supplementary Figure S1

sRAGE inhibits HMGB1 mRNA and protein expression in lung tissue (A) HMGB1 mRNA expression levels in lung tissue from mice in Control, Asthma, MSA-treated, and sRAGE-treated groups. Values represent the means ± SEM (n = 5–6). *P < 0.05 and **P < 0.01 compared with the Asthma group. (B) Representative images (upper panel) and quantification (lower panel) of western blot analysis for HMGB1 in lung tissue from mice in Control, Asthma, MSA-treated, and sRAGE-treated groups. Values represent the means ± SEM (n = 5–6). *P < 0.05 and **P < 0.01 compared with the Asthma group. To eliminate the possibility of sRAGE masking the HMGB1 antibody epitope, different doses of sRAGE (200 or 400 ng/ml) were directly added to the lung homogenates of asthmatic mice, and then HMGB1 expression was evaluated with the HMGB1 antibody by western blotting. The results showed that the addition of sRAGE to the lung homogenates had no effect on HMGB1 expression, suggesting that sRAGE did not mask the RAGE antibody epitope.

A

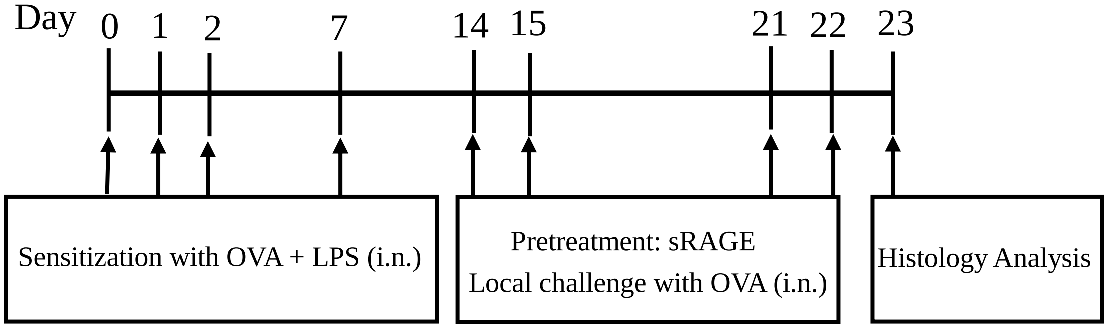

B

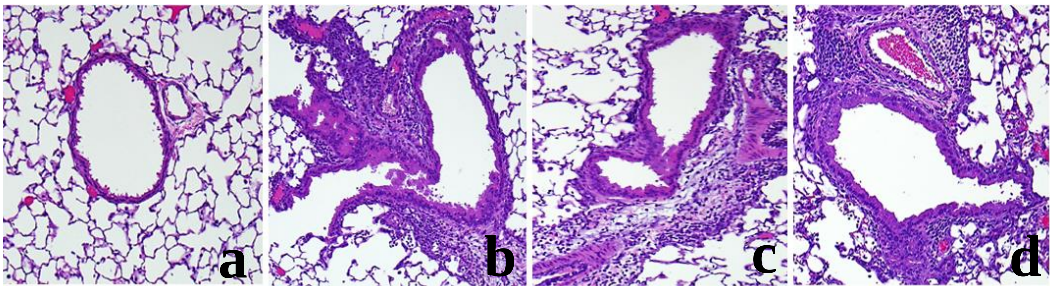

Supplementary Figure S2

Administration of sRAGE before allergen challenges has no effect on airway inflammation in asthmatic mice. (A) Protocols for the OVA and LPS-induced murine model of neutrophilic asthma and experimental intervention. Mice were intranasally sensitised with OVA plus LPS on days 0, 1, 2, and 7 and then challenged with OVA on days 14, 15, 21, and 22. sRAGE (200 or 400 μg/kg) was administered intranasally to mice at 30 min before the OVA challenges on days 14, 15, 21, and 22. One day after the final challenge, the mice were sacrificed for histological analysis. (B) Representative images of lung tissue histology (original magnification, ×200) in mice from Control (a), Asthma (b), sRAGE (200 μg/kg)-treated (c), and sRAGE (400 μg/kg)-treated groups (d) by H&E staining. Sensitised animals treated with sRAGE before

allergen challenge exhibited no effects on the appearance of inflammatory cell infiltration in lung tissue.

.

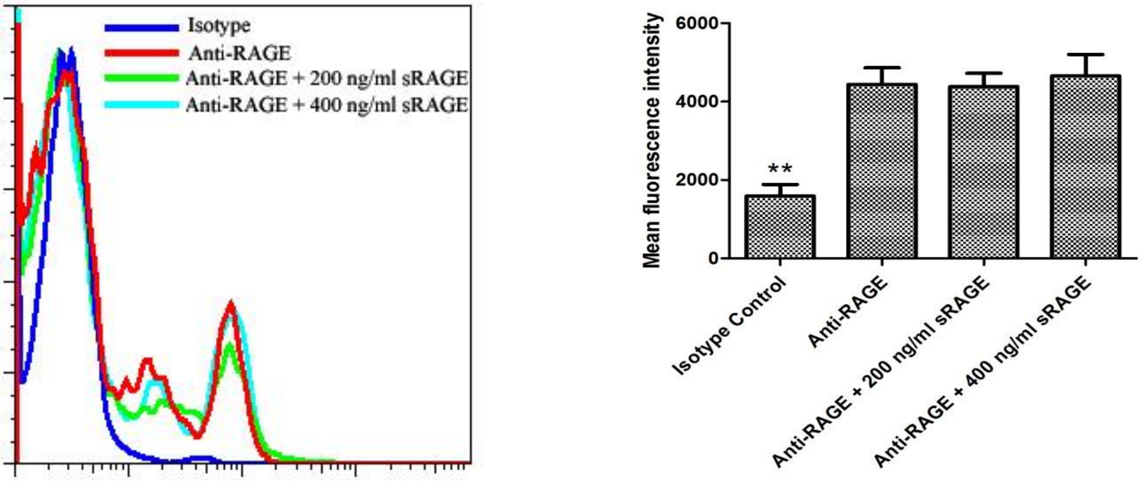

Supplementary Figure S3

Administration of sRAGE does not mask the RAGE antibody epitope. Representative images (left) and quantification (right) of flow cytometric analysis for RAGE expression in DCs from asthmatic mice. To eliminate the possibility of masking the RAGE antibody epitope, 200 or 400 ng/ml sRAGE was directly added to lung cell suspensions from asthmatic mice, and then RAGE expression in DCs was evaluated using the RAGE antibody by flow cytometry. Values represent the means ± SEM (n = 5~6). ***P* < 0.01 compared with the Asthma group. The results showed that administration of sRAGE had no effect on RAGE expression in DCs, suggesting that sRAGE does not mask the RAGE antibody epitope.

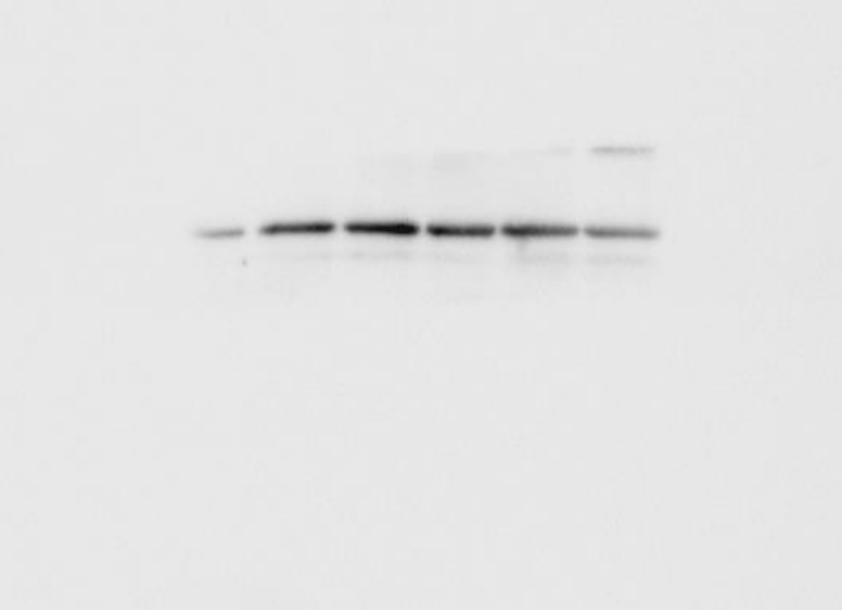

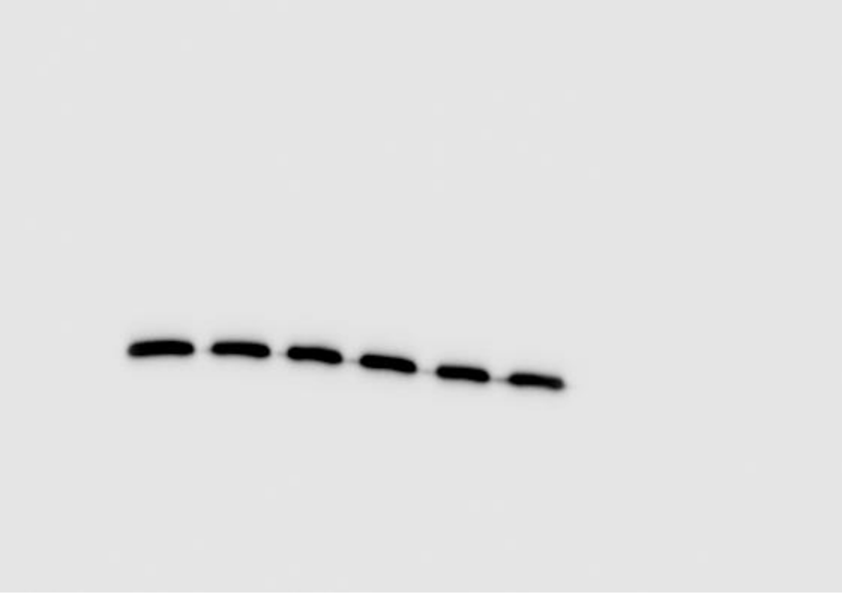

Supplement: Supplementary file 1 — Supplementary information [file 41598_2017_14667_MOESM1_ESM.pdf]
